# Caffeic Acid Phenethyl Ester Protects Kidney Mitochondria against Ischemia/Reperfusion Induced Injury in an *In Vivo* Rat Model

**DOI:** 10.3390/antiox10050747

**Published:** 2021-05-08

**Authors:** Justina Kamarauskaite, Rasa Baniene, Darius Trumbeckas, Arvydas Strazdauskas, Sonata Trumbeckaite

**Affiliations:** 1 Department of Pharmacognosy, Medical Academy, Lithuanian University of Health Sciences, Sukileliu Av. 13, LT-50162 Kaunas, Lithuania; sonata.trumbeckaite@lsmuni.lt; 2 Laboratory of Biopharmaceutical Research, Institute of Pharmaceutical Technologies, Lithuanian University of Health Sciences, Sukileliu Av. 13, LT-50162 Kaunas, Lithuania; 3 Neuroscience Institute, Lithuanian University of Health Sciences, Sukileliu Av. 13, LT-50162 Kaunas, Lithuania; rasa.baniene@lsmuni.lt (R.B.); arvydas.strazdauskas@lsmu.lt (A.S.); 4 Department of Biochemistry, Medical Academy, Lithuanian University of Health Sciences, Eiveniu Str. 4, LT-50161 Kaunas, Lithuania; 5 Department of Urology, Medical Academy, Lithuanian University of Health Sciences, Eivenių Str. 2, LT-50009 Kaunas, Lithuania; darius.trumbeckas@lsmu.lt

**Keywords:** kidney, caffeic acid phenethyl ester, antioxidant, mitochondria, ischemia/reperfusion, mitochondrial complexes, oxidative stress, reactive oxygen species

## Abstract

To improve ischemia/reperfusion tolerance, a lot of attention has been focused on natural antioxidants. Caffeic acid phenethyl ester (CAPE), an active component of the resinous exudates of the buds and young leaves of *Populus nigra* L., *Baccharis sarothroides* A., etc., and of propolis, possesses unique biological activities such as anti-inflammatory, antioxidant, immunomodulating, and cardioprotective effects, among others. There is a lack of studies showing a link between the antioxidant potential of CAPE and the mechanism of protective action of CAPE at the level of mitochondria, which produces the main energy for the basic functions of the cell. In the kidney, ischemia/reperfusion injury contributes to rapid kidney dysfunction and high mortality rates, and the search for biologically active protective compounds remains very actual. Therefore, the aim of this study was to identify the antioxidant potential of CAPE and to investigate whether CAPE can protect rat kidney mitochondria from in vivo kidney ischemia/reperfusion induced injury. We found that CAPE (1) possesses antioxidant activity (the reducing properties of CAPE are more pronounced than its antiradical properties); CAPE effectively reduces cytochrome c; (2) protects glutamate/malate oxidation and Complex I activity; (3) preserves the mitochondrial outer membrane from damage and from the release of cytochrome c; (4) inhibits reactive oxygen species (ROS) generation in the Complex II (SDH) F site; (5) diminishes ischemia/reperfusion-induced LDH release and protects from necrotic cell death; and (6) has no protective effects on succinate oxidation and on Complex II +III activity, but partially protects Complex II (SDH) from ischemia/reperfusion-induced damage. In summary, our study shows that caffeic acid phenethyl ester protects kidney mitochondrial oxidative phosphorylation and decreases ROS generation at Complex II in an in vivo ischemia/reperfusion model, and shows potential as a therapeutic agent for the development of pharmaceutical preparations against oxidative stress-related diseases.

## 1. Introduction

To improve ischemia/reperfusion tolerance, a lot of attention has recently been focused on natural antioxidants. Caffeic acid phenethyl ester (CAPE) is a natural phenolic compound, an active constituent of the resinous exudates of the buds and young leaves of *Populus nigra* L., *Baccharis sarothroides* A. Gray, *Cinnamomum cassia* L., *Rhodiola sacra*, *Melaleuca cajuput* Powell, etc., and of propolis [1]. CAPE possesses unique biological activities such as anti-inflammatory, antioxidant, immunomodulating cadioprotective, and neuroprotective effect, among others [1]. Moreover, CAPE has been successfully synthesized, which was inspired by the fact that the isolation and purification of CAPE from natural resources is expensive, low-yielding, and time-consuming [1].

Our recent in vitro study [2] has revealed that CAPE has a positive effect against ischemia-induced injury, namely that it partially protected rat kidney mitochondria from in vitro ischemia-induced damage. Since most studies, including our own, have been performed in vitro [2], the question remains open as to whether the same results will be observed in in vivo experiments. Such studies in kidney mitochondria have not yet been performed.

Ischemia/reperfusion injury is a critical condition that involves a series of events—disturbances of microcirculation; the development of cell inflammation; the generation of free radical, cytokine, chemokine, and leukocyte activation; and injury of the mitochondria and other ongoing processes [3]. Ischemic tissues need to recover blood supply for the regeneration of cells and to remove toxic metabolites. However, on the other hand, reperfusion of the ischemic tissue can paradoxically lead to much more serious damage than in the case of ischemia [4]. In the kidney, ischemia/reperfusion injury contributes to a pathological condition called acute kidney injury, which is a clinical syndrome involving rapid kidney dysfunction and high mortality rates [4].

It is well known that when organs are submitted to ischemia/reperfusion, the mitochondrial respiratory chain provokes the production of superoxide anion and other active forms of oxygen [5]. Reactive oxygen species (ROS), generated primarily in mitochondria, can initiate cellular response, which depends on ROS concentration. At the physiological level, ROS are involved in cell signaling pathways, whilst their high concentration promotes cellular damage and death [6]. Mitochondrial respiratory chain Complex I and Complex III were considered to be the main ROS producers, and Complex II (SDH)’s role in ROS synthesis was thought to be insignificant [7]. Complex II (SDH) consists of four subunits: SDHA—which contains of an active site with covalently bound FAD (flavine adenine dinucleotide, F site) and removes electrons from succinate; and SDHB—with three Fe-S clusters, which mediate electron transfer to ubiquinone located in the ubiquinone-binding site (Q site) formed by the SDHB, SDHC, and SDHD subunits. Subunits SDHC and SDHD of Complex II (SDH) are embedded in the mitochondrial inner membrane [8,9]. Recently, the importance of Complex II (SDH) was emphasized in the generation of ROS through reverse electron (RET) transport within Complex I. RET is important in ROS production mediated by Complex II (SDH) in the presence of a high concentration (more than 5 mM) of succinate. In this case, electrons received from Complex II (SDH) reduce the ubiquinone pool, reversely forced from ubiquinone to Complex I, and a large amount of ROS are formed [6]. Quinlan et al. showed that at a 0.5 mM concentration of succinate (low, physiological concentration of succinate), Complex II (SDH) can directly generate ROS, and for this, the flavin site of Complex II (SDH) is responsible [10]. Recently, interest in Complex II (SDH)-driven ROS generation has grown significantly when it was observed that Complex II (SDH) damage and/or functional loss directly and indirectly led to increased ROS production, i.e., via RET [6].

Antioxidant compounds are able to donate electrons to radicals, and can reduce them into more stable compounds. Therefore, the reducing capacity of a compound may serve as an indicator of its potential antioxidant activity. There are various studies showing the antioxidant potential of CAPE [11,12,13]; however, there is a lack of research showing a link between the antioxidant potential of CAPE and its mechanism of protective action on kidney mitochondrial function during in vivo ischemia/reperfusion.

Ischemia/reperfusion injury can be prevented or reduced by antioxidants [13] that decrease the toxic metabolites, which cause tissue damage. The main objective of this study was to identify the antioxidant (antiradical and reducing) potential of CAPE and to investigate whether CAPE can protect rat kidney mitochondria from in vivo ischemia/reperfusion-induced injury and ROS generation.

## 2. Materials and Methods

### 2.1. Animals and Experimental Model

Adult male Wistar rats (2–3 months old) weighing 200–250 g were housed under standard laboratory conditions and maintained on a natural light and dark cycle with free access to food and water. Animals were acclimatized to laboratory conditions before the experiment. Rats were injected with CAPE (22 mg/kg and 34 mg/kg of rat body) into the tail vein 90 min prior to ischemia/reperfusion. Warm kidney ischemia in rats was induced by adding clips around the kidney artery for 20 min, 30 min, 40 min, and 60 min (n = 3–5 in each group). Then, the clips were removed and reperfusion was performed for 30 min. After that, the kidneys were removed and washed free of blood in cold (4 °C) 0.9% KCl solution. Finally, the kidney tissue was used for the isolation of mitochondria.

### 2.2. Preparation of Isolated Kidney Mitochondria

The tissue was cut into small pieces, washed, and homogenized in a glass–Teflon homogenizer in a medium containing 250 mM sucrose, 10 mM Tris-HCl, and 1 mM EDTA (pH 7.3). The kidneys’ homogenate was centrifuged for 5 min at 750× *g* at 4 ℃. After centrifugation, the pellet was removed and the supernatants were centrifuged a second time at 10,000× *g* for 10 min at 4 ℃. The mitochondrial pellet was resuspended in the same buffer and stored on ice.The protein concentration was determined by the Biuret method using bovine serum albumin as a standard [14].

### 2.3. Measurement of Mitochondrial Respiration

The mitochondrial functions were measured using high resolution respirometry system Oxygraph-2k, glutamate/malate and succinate were used as substrates. Incubation medium contained 150 mM KCl, 10 mM Tris-HCl, 5 mM KH_2_PO_4_, and 1 mM MgCl_2_ × 6H_2_O (pH 7.2). Routine respiration rate was measured by adding mitochondria (0.25 mg/mL) and respiratory substrates: 5 mM glutamate + 5 mM malate or 15 mM succinate (+50 nM rotenone). Then, 1 mM ADP was added in order to measure mitochondrial state 3 respiration rate followed by the addition of exogenous cytochrome c (32 µM) in order to determine the mitochondrial outer membrane damage. Mitochondrial respiration rates were expressed as pmol O/s/0.25 mg protein.

### 2.4. Measurement of Antioxidant Activity of CAPE

ABTS method: 3 mL of diluted ABTS working solution produced by reacting 7 mM ABTS aqueous solution with 2.45 mM potassium persulfate was mixed with 10 µL of the CAPE or Trolox. A decrease in absorbance was measured at 734 nm using a double beam UV/VIS spectrophotometer M550 (Spectronic CamSpec, Garforth, UK) [15].

DPPH method: 3 mL of DPPH working solution (60 µM/L in 96.3% ethanol) was mixed with 10 µL of the CAPE or Trolox. A decrease in absorbance was measured at 517 nm [15].

FRAP method: 3 mL freshly prepared FRAP reagent, consisting of 300 mM acetate buffer, 10 mM TPTZ in 40 mM HCl, and 20 mM iron (III) chloride in a final ratio of 10:1:1 (*v*/*v*/*v*) was mixed with 10 µL of the CAPE or Trolox. An increase in absorbance was recorded at 593 nm [16].

CUPRAC method: 3 mL freshly prepared CUPRAC reagent, consisting of 0.01 M copper (II) chloride, 0.001 M ammonium acetate buffer solution, and 0.0075 M neocuproine in a final ratio of 1:1:1 (*v*/*v*/*v*) was mixed with 10 µL of the CAPE or Trolox. An increase in absorbance was recorded at 450 nm [17].

The obtained absorption of five different concentrations of tested CAPE and Trolox were used to construct calibration curves. Scavenging and reducing activities of CAPE were expressed as TEAC (Trolox equivalent antioxidant capacity) and calculated using the following equation [16,18], where slope (a) from calibration curve (y = ax + b) was used to calculate the TEAC value:

$$\mathrm{TEAC}=\frac{a_{\mathrm{sample}}}{a_{\mathrm{trolox}}}$$

### 2.5. Measurement of Cytochrome c Reduction Level of CAPE

The reduction of cytochrome c was recorded with a Helios Gama spectrophotometer in 1 mL of incubation medium and taken as the experimental blanks. Then, cytochrome c (20 µM) was added to the same buffer and the cytochrome c spectrum was recorded over the range 500–600 nm (cytochrome c reduction maximum was taken as 550 nm). After that, CAPE (in the concentration range of 5 µM, 10 µM, and 15 µM) were added and registered every 3 min (registration duration up to 21 min). The absorption peak height at 550 nm was taken and compared to the absorption peak of completely reduced cytochrome c at the end of the test by adding a few crystals of dithionite (sodium hydrosulfite). The absorption peak of dithionite-reduced cytochrome c was equated to 100% of reduced cytochrome c. All measurements were performed at room temperature.

### 2.6. Measurement of Mitochondrial Respiratory Chain Complex I, II+III, II (SDH)

The activity of Complex I was measured spectrophotometrically at 340 nm, according to the kinetics of NADH oxidation. The activity of Complex II+III in mitochondria was measured following the reduction of cytochrome c at 550 nm (after the addition of succinate, respectively) using an extinction coefficient for cytochrome c of 21.1/mM/cm, as described in [19]. Complex II (SDH) activity was measured at 600 nm following the reduction of 2,6-dichlorophenolindophenol using an extinction coefficient of 19.1/mM/cm.

### 2.7. Measurement of H_2_O_2_ Generation in Kidney Mitochondria

The generation of H_2_O_2_ in the kidney mitochondria was determined fluorimetrically (fluorometer, Thermo Scientific) with excitation at 544 nm and emission at 590 nm. Mitochondria, 0.05 mg/mL, were incubated in assay medium containing 150 mM KCl, 10 mM Tris-HCl, 5 mM KH_2_PO_4_, and 1 mM MgCl_2_ at 37 °C (pH 7.2). Complex II (SDH) substrate succinate—0.4 mM (low, physiological concentration) or 5 mM (high, saturating concentration)—were added to the appropriate wells. Electron transfer chain inhibitors were added to the appropriate wells: Complex I inhibitor rotenone (1 mM), complex II (SDH) F center (flavin center) inhibitor malonate (0.2 M), Complex II (SDH) Q center (ubiquinone center) inhibitor atpenin A5 (2 mM), and Complex III inhibitor myxothiazole (2 µM). Then, 2 U/mL horseradish peroxidase and 10 mM Amplex Red dye were added to each well. The fluorescence signal was calibrated using known amounts of H_2_O_2_.

### 2.8. Measurement of Lactate Dehydrogenase Activity

Lactate dehydrogenase (LDH) activity in the cytosolic fractions was measured spectrophotometrically according to the NADH oxidation rate at 340 nm, as described in [20].

### 2.9. Statistical Analysis

Data are presented as mean ± SEM of 3–5 separate experiments and were analyzed using the software package SPSS 25.0 software (Chicago, IL, USA). The mean for individual experiments was obtained from at least three repeated measurements. Statistical analysis was performed by applying the one-way ANOVA method and the two-way ANOVA (data of effect of CAPE on cytochrome c reduction), followed by the Fisher LSD post hoc test for means separation. Differences between the two groups (data of antioxidant activity of CAPE in vitro) were analyzed by Student’s *t*-test. *p* values < 0.05 were considered statistically significant.

## 3. Results

### 3.1. Evaluation of Antioxidant (Antiradical and Reducing) Activity of CAPE

**Figure 1:**
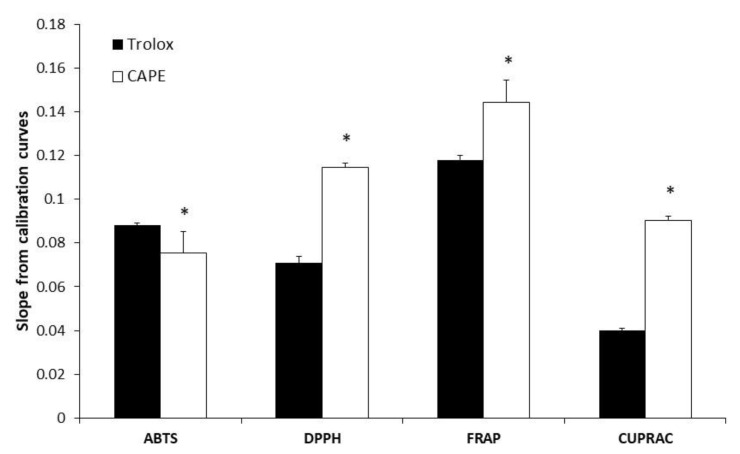
Antioxidant activity of CAPE in vitro. Antioxidant activity was measured, as described in the Materials and Methods section, by ABTS, DPPH, FRAP, and CUPRAC assays. The slope (a) from the calibration curve (y = ax + b) was used to calculate the TEAC value. * *p* < 0.05 vs. Trolox. Student’s *t*-test was used to compare the groups.

For the purpose of better understanding of the structure–antioxidant activity relationship, we investigated CAPE antioxidant activity by four different methods: ABTS, DPPH, CUPRAC, and FRAP. All calibration curve equations followed a linear regression model (*p* < 0.05) (Table A1 and Table A2 in Appendix A). The Trolox equivalent antioxidant capacity values (TEAC) indicated how many times the radical scavenging and reducing activities of the tested antioxidant were greater than that of Trolox. TEAC_ABTS or DPPH or FRAP or CUPRAC_ values were determined for CAPE (Table 1). The higher the slope of the calibration curve of an individual phenolic compound, the stronger the antioxidant properties of the compound (Figure 1). CAPE scavenging activity by the ABTS method (TEAC_ABTS_) was 0.85 ± 0.07 (Table 1), and this showed that CAPE scavenging activity was only 1.2 times lower than Trolox. The DPPH method (TEAC_DPPH_) showed that CAPE scavenging activity was 1.65 ± 0.09, which was greater than that of Trolox. This result shows that CAPE has good scavenging capabilities for DPPH^●^. The DPPH method, according to the mechanism of action, reveals not only the antiradical properties of the compound, but also its reducing properties. Significantly, the greatest TEAC_FRAP_ and TEAC_CUPRAC_ values were determined for CAPE (Table 1). The reducing activity of CAPE was 1.22 ± 0.03 and 2.24 ± 0.05 times (detected by the FRAP and CUPRAC methods, respectively) greater than Trolox (Figure 1, Table 1). These results demonstrate that CAPE has a strong potency to donate electrons to reactive free radicals, converting them into more stable forms. The results obtained show that CAPE possesses both radical scavenging and reducing activities. The reducing activity of CAPE was more pronounced than the radical scavenging activity because the ratio between TEAC_ABTS_ and TEAC_FRAP_ or TEAC_DPPH_ and TEAC_CUPRAC_ was less than 1.

### 3.2. Measurement of Cytochrome c Reduction Level of CAPE

**Figure A1:**
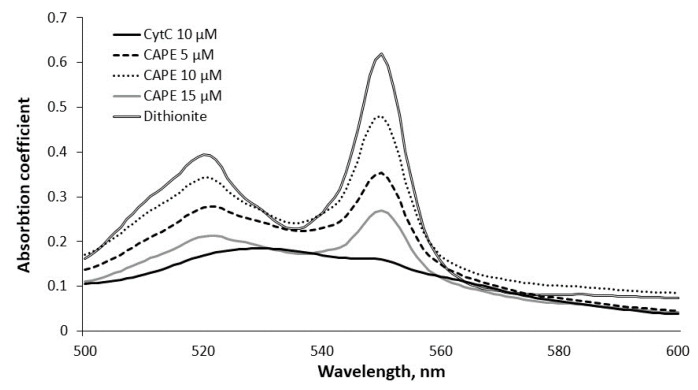
Original spectra of cytochrome c (oxidized) reduced by caffeic acid phenethyl ester.

First, we determined the capacity of CAPE to reduce cytochrome c (directly in vitro). The absorbance spectrum of cytochrome c was measured in the range of 500–600 nm over time in the presence of various (5 µM, 10 µM, and 15 µM) concentrations of CAPE. The added cytochrome c was in the oxidized form, and there was no change over the course of the experiment (Figure A1). The typical curve, showing the capacity of CAPE to reduce cytochrome c, is shown in Figure A1. Dithionite served as a control (reduced cytochrome c to 100%).

**Figure 2:**
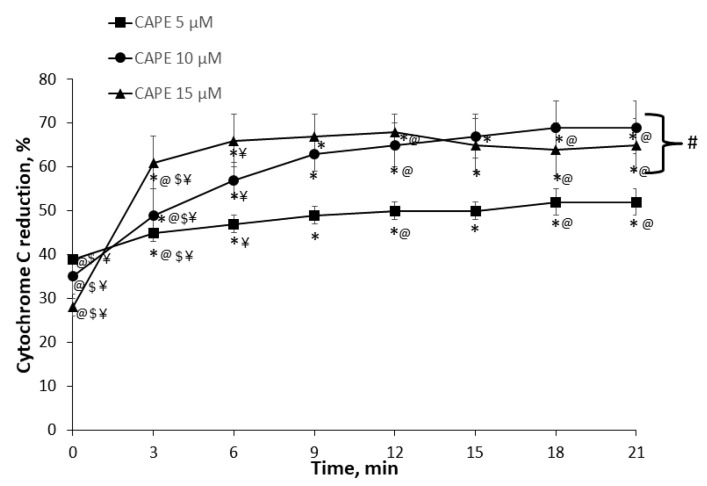
Effect of caffeic acid phenethyl ester on cytochrome c reduction. * *p* < 0.05 vs. time at the 0 min point; @ *p* < 0.05 vs. time at the 6 min point; $ *p* < 0.05 vs. time at the 9 min point; ¥ *p* < 0.05 vs. time at the 21 min point; # *p* < 0.05 vs. CAPE at a 5 µM concentration. Two-way ANOVA followed by a Fisher’s LSD post hoc test were used to compare the effects.

We revealed that CAPE is a potent cytochrome c reducing compound. In the presence of 15 µM CAPE, there was fast and almost complete reduction of cytochrome c within 6–9 min of incubation; during the first minute it was 28 ± 2%, reaching 66 ± 6% after 6 min. The maximal reduction was achieved after 9 min (67 ± 5%) (Figure 2). The reducing capacity of 5 µM CAPE increased from 39 ± 1% during the first minute to 47 ± 2% (at 6 min), and reached the maximal reducing capacity (69 ± 6%) at 21 min. 10 µM CAPE reduced cytochrome c to 35 ± 4% within the first minute, then increased to 57 ± 4% at 6 min, and the maximum (67 ± 5%) was reached at 21 min. Thus, the lower capacity to reduce cytochrome c had the lowest concentration (5 µM) of CAPE (57 ± 4%), whereas 10 µM and 15 µM of CAPE were similarly effective (reduced to 67–69%). Thus, our results show the high potential of CAPE to reduce cytochrome c.

### 3.3. Effects of Ischemia/Reperfusion and Pretreatment with CAPE on Kidney Mitochondrial Respiration Rates

**Figure 3:**
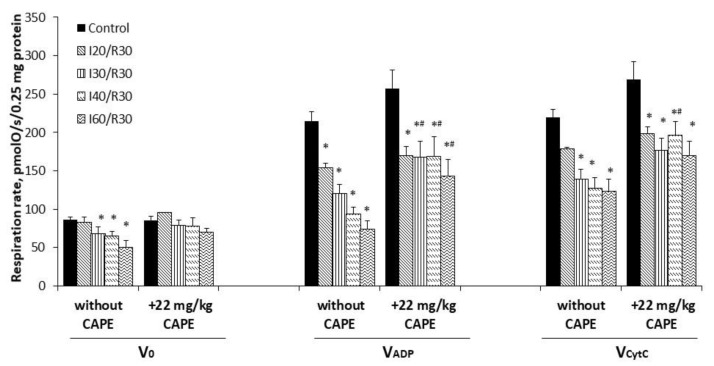
Effect of ischemia/reperfusion and CAPE on mitochondrial routine and state 3 respiration rate with glutamate/malate as substrates. Mitochondrial respiration rate was measured, as described in the Materials and Methods section, using 5 mM glutamate + 5 mM malate as substrates. V_0_—routine respiration rate in the presence of 0.25 mg/mL of the mitochondria and substrates; V_ADP_—state 3 respiration rate in the presence of 1 mM ADP; V_CytC_—state 3 respiration rate in the presence of 32 µM cytochrome c; * *p* < 0.05 vs. the respective control; # *p* < 0.05 vs. ischemia/reperfusion without CAPE. One-way ANOVA followed by a Fisher’s LSD post hoc test were used to compare the effects.

To investigate if CAPE protects mitochondria from ischemia-induced mitochondrial damage, CAPE was injected 90 min before the onset of ischemia (20, 30, 40, or 60 min) followed by 30 min of reperfusion. As can be seen from Figure 3, ischemia/reperfusion alone (starting from 20 min of ischemia/reperfusion) caused significant injury of the mitochondrial function (state 3 respiration decreased by 28–66% with glutamate/malate as a substrate, whereas, with succinate, mitochondrial function was affected later (i.e., after 30 min ischemia/reperfusion) by 42–45%, *p* < 0.05) (Figure 5).

**Figure 4:**
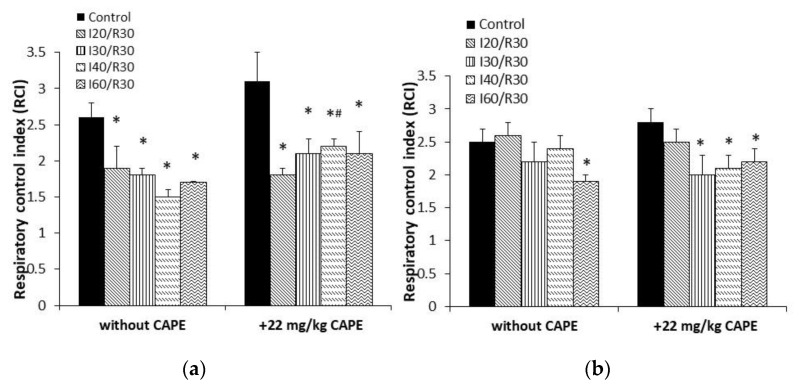
Effect of ischemia/reperfusion and CAPE on the mitochondrial respiratory control index (RCI). Measurements were performed in the presence of 5 mM glutamate + 5 mM malate (**a**) or 15 mM succinate (+50 nM rotenone) (**b**) as substrates. Mitochondrial respiratory control index (RCI) is taken as the ratio between the oxygen uptake rates in state 3 and the routine respiration rate (RCI = V_3_/V_0_). Each column represents the mean ± SEM of four independent experiments; *p < 0.05 vs. the respective control; # *p* < 0.05 vs. ischemia/reperfusion without CAPE. One-way ANOVA followed by a Fisher’s LSD post hoc test were used to compare the effects.

**Figure 5:**
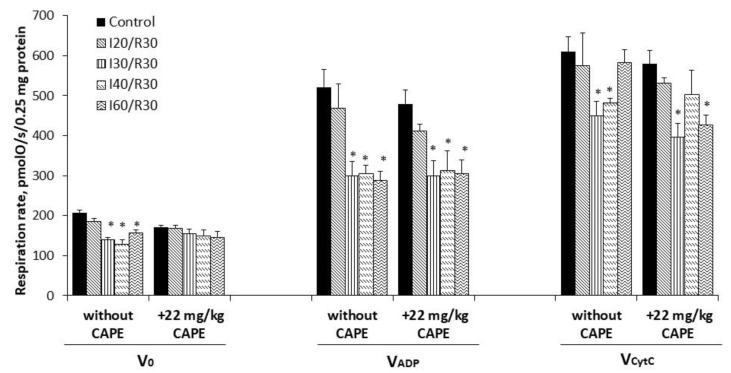
Effect of ischemia/reperfusion and CAPE on the kidney mitochondrial routine and state 3 respiration rate with succinate as a substrate. Mitochondrial respiration rate was measured, as described in the Materials and Methods section, using 15 mM succinate (+50 nM rotenone) as a substrate. V_0_—routine respiration rate in the presence of 0.25 mg/mL of mitochondria and substrate; V_ADP_—state 3 respiration rate in the presence of 1 mM ADP; V_CytC_—state 3 respiration rate in the presence of 32 µM cytochrome c, * *p* < 0.05 vs. the respective control. One-way ANOVA followed by a Fisher’s LSD post hoc test were used to compare the effects.

Pretreatment of rats with CAPE (22 mg/kg of rat body) alleviated the ischemia-induced damage of the mitochondria (Figure 3). Thus, the mitochondrial state 3 respiration with glutamate/malate increased from 120 ± 12 pmolO/s/0.25 mg (untreated group, I30/R30) to 168 ± 21 and 177 ± 4 pmolO/s/0.25 mg (+CAPE), thus, by 1.40-fold. When CAPE was applied before the onset of 40 min and 60 min of ischemia/reperfusion, it was increased by 2.3-fold and 1.9-fold, respectively. Routine respiration rate after 20 min, 30 min, 40 min, and 60 min of ischemia/30 min of reperfusion after pretreatment with CAPE was practically restored to the control level (substrate glutamate/malate) (Figure 3). The respiratory control index increased from 1.5 and 1.7 (untreated group) to 2.2 after pretreatment with CAPE, Figure 4a. It is important to note that CAPE had no statistically significant protective effect on the succinate oxidation (Figure 5) or respiratory control index (Figure 4b).

**Figure 6:**
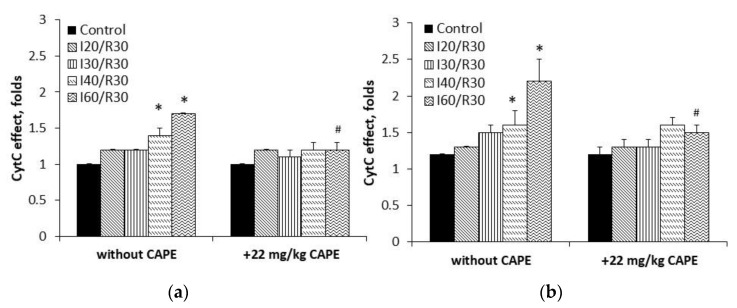
Effect of ischemia/reperfusion and CAPE on cytochrome c effect. Measurements were performed in the presence of 5 mM glutamate + 5 mM malate (**a**) or 15 mM succinate (+ 50 nM rotenone) as substrates (**b**). Cytochrome c effect (Cyt c effect) was calculated as the ratio between oxygen uptake rates in state 3 in the presence of cytochrome c and state 3 (CytC effect = V_CytC_/V_ADP_). * *p* < 0.05 vs. the respective control; # *p* < 0.05 vs. ischemia/reperfusion without CAPE. One-way ANOVA followed by a Fisher’s LSD post hoc test were used to compare the effects.

Moreover, the cytochrome c effect obviously increased due to ischemia/reperfusion damage; however, it was clearly reduced after pretreatment with CAPE (for comparison, cytochrome c effect (substrate glutamate/malate) after ischemia/reperfusion varied between 1.20–1.70-fold), whereas after pretreatment with CAPE, it decreased to 1.0–1.2-fold (Figure 6a). Analyzing succinate oxidation, the cytochrome c effect after 30–60 min of ischemia /reperfusion varied between 1.5–2.2-fold, whereas after pretreatment with CAPE, it decreased to 1.3–1.6-fold (Figure 6b).

Similar protective effects on kidney mitochondrial function were obtained when a higher dose of CAPE (34 mg/kg of rat body) was used (data not shown).

### 3.4. Effects of Ischemia/Reperfusion and Pretreatment with CAPE on Kidney Mitochondrial Complex I, II+III, and II (SDH) Activity

**Figure 7:**
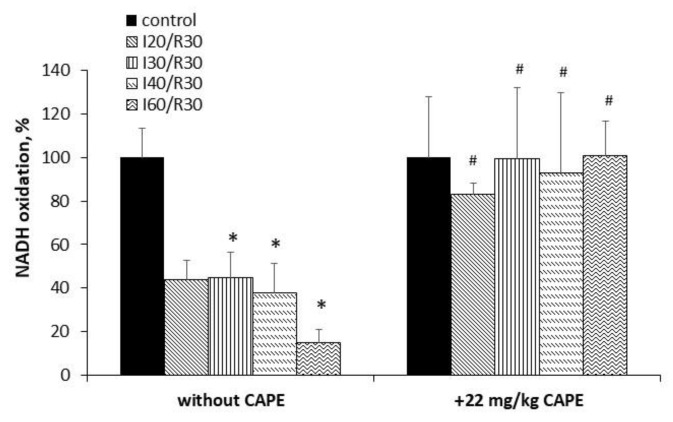
Effect of ischemia/reperfusion and CAPE on Complex I activity in the kidney mitochondria. The Complex I activity was measured spectrophotometrically at 340 nm, as described in the Materials and Methods section. * *p* < 0.05 vs. the respective control; # *p* < 0.05 vs. ischemia/reperfusion without CAPE. One-way ANOVA followed by a Fisher LSD post hoc test was used to compare the effects.

Since our data have revealed the protective effect of CAPE on glutamate/malate oxidation, we first checked whether CAPE can increase ischemia/reperfusion-reduced mitochondrial Complex I activity, which was obviously diminished (by 33%, 56%, 62%, and 85 % after ischemia for 20 min, 30 min, 40 min, and 60 min/reperfusion, respectively, *p* < 0.05, Figure 7). Pretreatment with CAPE (22 mg/kg of rat body) fully restored the activity of Complex I to control levels (Figure 7), *p* < 0.05.

**Figure 8:**
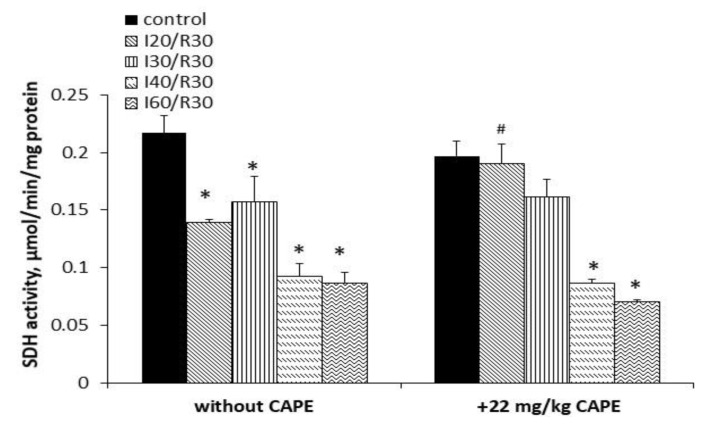
Effect of ischemia/reperfusion and CAPE on SDH (Complex II) activity in the kidney mitochondria. The SDH activity was measured spectrophotometrically at 600 nm, as described in the Materials and Methods section. * *p* < 0.05 vs. the respective control; # *p* < 0.05 vs. ischemia/reperfusion without CAPE. One-way ANOVA followed by a Fisher’s LSD post hoc test were used to compare the effects.

Ischemia/reperfusion alone diminished Complex II (SDH) activity by 36%, 27%, 57%, and 60% after 20 min, 30 min, 40 min, and 60 min of ischemia/reperfusion, respectively, *p* < 0.05. CAPE protected the activity of Complex II (SDH) after 20 min and 30 min of ischemia/reperfusion by 32% and 10%, respectively, *p* < 0.05; however, it was no more effective after longer (40 min and 60 min) periods of ischemia/reperfusion, where the Complex II (SDH) activity had decreased obviously (Figure 8).

**Figure 9:**
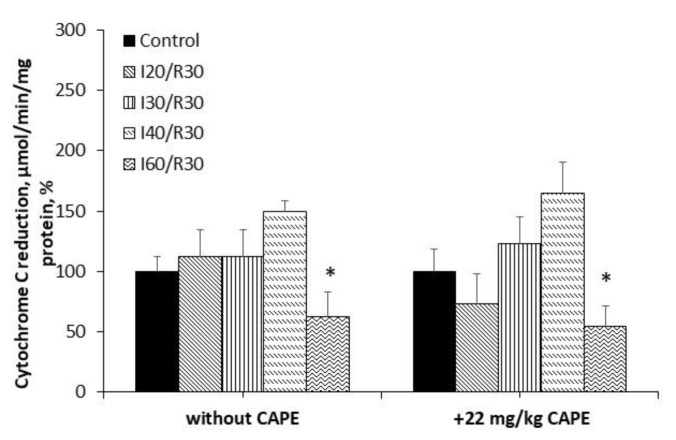
Effect of ischemia/reperfusion and CAPE on Complex II+III activity in the kidney mitochondria. The Complex II+III activity was measured spectrophotometrically at 550 nm, as described in the Materials and Methods section. *p < 0.05 vs. the respective control. One-way ANOVA followed by a Fisher’s LSD post hoc test were used to compare the effects.

The activity of Complex II+III was unchanged during 20–40 min of ischemia/reperfusion; however, it statistically significantly decreased (by 38%) after a long period (60 min) of ischemia/reperfusion, *p* < 0.05 (Figure 9). CAPE had no protective effect on Complex II+III activity after 60 min of ischemia/reperfusion (it remained decreased by 45%), *p* < 0.05.

### 3.5. Effects of CAPE on Complex II Mediated ROS Generation in Kidney Mitochondria after Ischemia/Reperfusion

**Figure 10:**
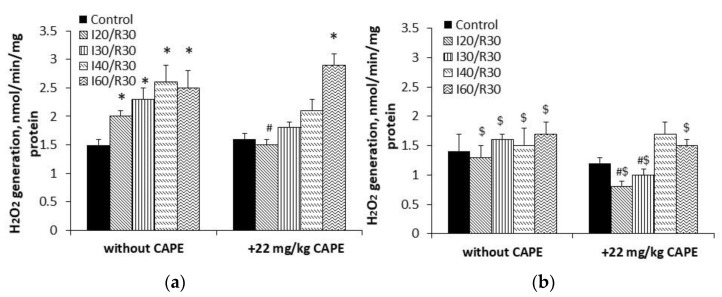
Effect of ischemia/reperfusion and CAPE on H_2_O_2_ generation in the kidney mitochondria. The generation of H_2_O_2_ in the kidney mitochondria was determined fluorimetrically, as described in the Materials and Methods section (excitation at 544 nm, emission at 590 nm), in the presence of the Complex I inhibitor rotenone (5 μM) and the Complex III inhibitor myxothiazole (2 μM). Substrates of succinate 0.4 mM (**a**) and succinate 5 mM (**b**). * *p* < 0.05 vs. the respective control; # *p* < 0.05 vs. the group without CAPE; $ *p* < 0.05 vs. the 0.4 mM succinate group. One-way ANOVA followed by a Fisher’s LSD post hoc test were used to compare the effects.

**Figure 11:**
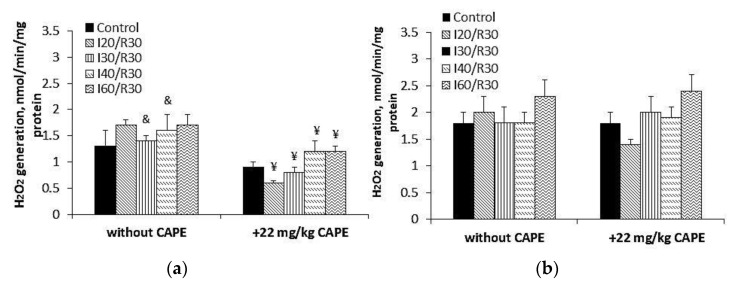
Effect of ischemia and CAPE on H_2_O_2_ generation in the kidney mitochondria. The generation of H_2_O_2_ in the kidney mitochondria was determined fluorimetrically, as described in the Materials and Methods section (excitation at 544 nm, emission at 590 nm), in the presence of 0.4 mM substrate succinate, the Complex I inhibitor rotenone (5 μM), and the Complex III inhibitor myxothiazole (2 μM). Complex II (SDH)-specific F and Q site inhibitors were added to the appropriate wells: The F site inhibitor malonate (**a**) and the Q site inhibitor atpenin A5 (**b**). & *p* < 0.05 vs. the 0.4 mM succinate group in the presence of the Complex I inhibitor rotenone (5 μM) and the Complex III inhibitor myxothiazole (2 μM) (Figure 12a, without CAPE); ¥ *p* < 0.05 vs. the group without CAPE. One-way ANOVA followed by a Fisher’s LSD post hoc test were used to compare the effects.

To assess the effect of CAPE on Complex II (SDH)-driven ROS generation in the kidney mitochondria after ischemia/reperfusion, we measured H_2_O_2_ synthesis in the kidney mitochondria in the presence of the Complex I inhibitor rotenone and the Complex III inhibitor myxothiazol. Our results (Figure 10) demonstrated that, at 0.4 mM succinate concentration, ischemia (20, 30, 40, 60 min)/reperfusion (30 min) induced an ischemia time-dependent increase in H_2_O_2_ generation by 32%, 52%, 70%, and 59%, respectively (Figure 10a, *p* < 0.05). Pretreatment of rats with CAPE affected the ability of Complex II (SDH) to generate H_2_O_2_. H_2_O_2_ synthesis after 20, 30, and 40 min of ischemia/30 min reperfusion decreased and returned to the control level (Figure 10a). At saturating (5 mM) succinate concentration, H_2_O_2_ generation after ischemia (20, 30, 40, 60 min)/reperfusion (30 min) was 38%, 34%, 43%, and 30% lower as compared with 0.4 mM succinate concentration (Figure 10a,b, *p* < 0.05). Neither ischemia/reperfusion nor the pretreatment of rats with CAPE had an effect on Complex II (SDH)-driven H_2_O_2_ generation (Figure 10b). To find out which active site of Complex II (SDH) is involved in ROS synthesis after ischemia/reperfusion, we inhibited the FAD binding site of Complex II (SDH) (F site) with malonate and the Q binding site (Q site) with atpenin A5 (Figure 11). The results (Figure 11a) showed that malonate inhibited ROS generation at the F site after 30 and 40 min of ischemia/reperfusion by about 40% as compared with the rotenone and myxothiazol groups, *p* < 0.05, respectively (Figure 10a). Atpenin A5 (Figure 11b) had no effect on the Q site ROS generation after ischemia/reperfusion. CAPE-inhibited ROS generation at the F site (Figure 11a) in the control and the ischemia/reperfusion affected the mitochondria by 43–59%, and had no effect on the Q site ROS generation after ischemia/reperfusion.

### 3.6. Effects of CAPE on LDH Activity after Ischemia/Reperfusion

Our results (Table 2) demonstrated that ischemia (20, 30, 40, 60 min)/reperfusion 30 min induced damage of the kidney cells and, as a consequence of that, the glycolysis enzyme lactate dehydrogenase (LDH) was released. LDH activity in the cytosolic fractions decreased by about 20% after ischemia (20, 30, 40, 60 min)/reperfusion 30 min as compared to control group. Pretreatment with CAPE (22 mg/kg) protected the kidney from necrotic cell death after 20, 30, and 40 min of ischemia/30 min reperfusion (Table 2), and completely restored the activity of LDH.

## 4. Discussion

Mitochondria provide over 90% of the ATP required for cell metabolism and are involved in the pathogenesis of human diseases and other injuries such as kidney ischemia/reperfusion [21]. Ischemia/reperfusion is a pathological condition caused by an initial restriction of the blood supply to an organ, followed by the subsequent restoration of perfusion and the concomitant reoxygenation [22]. For improving kidney ischemia/reperfusion tolerance, much attention has recently been focused on antioxidants. One such promising antioxidant, the interest in which is growing, is CAPE. This compound may play a significant protective role in various disease conditions [23].

The dose of CAPE (22 mg/kg or 34 mg/kg) was chosen based on the data in the literature, where the dose ranged from 1 mg/kg to 40 mg/kg [24,25,26]. The authors have revealed that CAPE at these doses ameliorates ischemia/reperfusion-induced damage in brain or intestinal tissue [24,25,26]. According to Akyol et al., CAPE distributes into tissues extensively [27], and after i/v administration, the elimination half-life of CAPE ranges between 21.2–26.7 min [27].

Thus, by using four various methods (antiradical—ABTS and DPPH, and reducing—FRAP and CUPRAC), we revealed that CAPE has strong antioxidant potential (both radical scavenging and reducing activities). It is important to note that the reducing activity of CAPE was more pronounced than its radical scavenging activity. This explains the possible mechanisms of the antioxidant effect: CAPE is able to donate an electron to free radicals, making radicals stable. In agreement with our data, Göçer et al. also showed [28] that CAPE had good scavenging capabilities for DPPH^∙^, ABTS^∙^^+^, DMPD^∙^^+^, and O_2_^∙^^−^ radicals, and good reducing capacity. CAPE’s reducing properties were higher than those of α-tocopherol and Trolox [28]. According to them, CAPE had a potent scavenging effect on DPPH radicals. higher than that of abovementioned antioxidants. We found that the reducing power of CAPE by FRAP and CUPRAC assays was higher than that of Trolox (Fe^3+^ reduction into Fe^2+^ showed electron-donating activity, and this may explain the mechanism of antioxidant action of CAPE, and the cupric ion (Cu^2+^) reducing power of CAPE by the CUPRAC method was also higher than that of Trolox). Our results also showed that CAPE has an effective scavenging effect on DPPH^∙^ radicals; however, CAPE’s scavenging capability for ABTS^∙^^+^ radicals was lower than that of Trolox. The antioxidant activities of CAPE are explained by the structure of CAPE: catechol fragments offer the compound strong antioxidant properties. The presence of ortho-dihydroxyl functionality in the catechol ring allows for antiradical activity [29]. CAPE is a hydroxyl derivative of cinnamic acid, and the existence of the CH_2_=CH–COOH group in cinnamic acids provides higher antioxidant activity compared to other phenolic acids [30]. Esters of phenolic acids may be more active than free phenolic acids because the ester group better stabilizes the radical forms [31]. An additional side chain between the benzene ring and the ester group with a double bond also has a positive effect on the activity of CAPE. More pronounced reducing activity explains the possible mechanisms of the antioxidant effect: CAPE is able to donate an electron to free radicals, making radicals stable.

Several studies indicated that CAPE plays an important protective role against ischemia/reperfusion injury in multiple target tissues, including the brain, retina, heart, skeletal muscles, testis, ovaries, intestine, colon, and liver [30,32]. It was demonstrated by Celik et al. that exogenously administered CAPE (20 µM/ kg) effectively protected the ovary tissues against oxidative damage [33]. The protective effects are explained by them leading a decrease in malondialdehyde (MDA) and xanthine oxidase (XO), and an increase in glutathione (GSH). They concluded that CAPE could be useful in treating ovarian ischemia/reperfusion injury and possibly other clinical conditions involving excess free radical production [33]. According to their biochemical and histopathological findings, Irmak et al. demonstrated that the prophylactic peritoneal administration of CAPE (10 µM/kg from 25 µM/mL solution) protects the kidneys from reperfusion injuries more than α-tocopherol (vitamin E). CAPE prevents kidney ischemia/reperfusion injury through the inhibition of lipid peroxidation, and authors suggest that the parenteral administration (intravenous or intraperitoneal) of CAPE would be helpful in humans undergoing reconstructive kidney clinical conditions [34]. Teke et al. concluded that CAPE provides beneficial actions against intestinal mucosal injury mediated by ischemia/reperfusion of the small intestine. They guess that such effects of CAPE are related to its anti-inflammatory and antioxidant properties [35].

As ischemia/reperfusion damage is associated with the burst of ROS, we also checked the protective effects of CAPE on ROS generation mediated by Complex II (SDH). We revealed that ROS generation is clearly increased during ischemia/reperfusion when low (0.4 mM), i.e., physiological succinate concentration, was used. CAPE powerfully diminishes ROS generation (Figure 10a) mediated by Complex II (SDH) in the presence of rotenone and myxothiazol. However, at a saturating succinate concentration (5 mM), neither ischemia/reperfusion nor CAPE had an effect on ROS generation. These results are in line with other authors, who showed that a high concentration of succinate (more than 5 mM) may suppress ROS production at the flavine site in Complex II (SDH) [10]. To find out which active site of Complex II (SDH) is involved in ROS synthesis after ischemia/reperfusion, we inhibited the FAD binding site of Complex II (F site) with malonate, and found that the ability of this site to participate in ROS generation was inhibited after 30 and 40 min of ischemia with 30 min reperfusion; however, malonate had no effect on ROS after prolonged ischemia/reperfusion (Figure 10a and Figure 11a). Complex II (SDH) Q site inhibition with atpenin A5 (Figure 11b) had no effect on ROS generation after ischemia/reperfusion; in fact, ROS generation at this site was higher than at the F site. Siebels and Drose, using submitochondrial particles, showed that atpenin A5 increased ROS generation at this site [36]. Grivennikova et al. [37] showed that, in Complex II, (SDH) the Fe-S ([3Fe-4S]) center is the site of superoxide generation, and that superoxide is rapidly and irreversibly eliminated by cytochrome c. Guzy et al. showed that defects in the SDHB, SDHC, or SDHD subunits of Complex II (SDH), but not the SDHA subunit, caused an increase in ROS production mediated by Complex II (SDH) and lead to tumorigenesis [38,39,40]. Our results showed that Complex II (SDH) activity after 30 and 40 min of ischemia/reperfusion decreased significantly by 27% and 57%, respectively, along with decreased mitochondrial respiration by about 40% during these periods of ischemia. CAPE pretreatment protected Complex II (SDH) activity and inhibited ROS generation at the Complex II F site in the control, and ischemia/reperfusion affected mitochondria by 43–59%; however, CAPE had no effect on the Q site ROS generation (Figure 11). It was mentioned that mitochondrial ROS production is involved in ischemia/reperfusion injury, and the Complex II (SDH) inhibitors atpenin A5, malonate, or the new mitochondria-targeted Complex II (SDH) inhibitor tanshinone IIA exert protective effects in different cases of ischemia/reperfusion [41,42,43]. Wang et al. showed that a new ferulic acid derivative is protective in ischemia/reperfusion oxidative injury [44].

Our results showed that CAPE suppresses the release of cytochrome c from the mitochondria by protecting the stability of the mitochondrial outer membrane. Cytochrome c, released from the mitochondria into the cytosol, triggers the formation of the apoptosome, resulting in the activation of caspases [45]. Moreover, we found that CAPE effectively reduces cytochrome c. The redox state of cytochrome c may regulate apoptosis—the reduced form (Fe^2+^) of cytochrome c cannot induce apoptosis, while the oxidized form of cytochrome c (Fe^3+^) can induce apoptosis. These results correlate with the results obtained by the FRAP assay, which showed that CAPE has a strong ability to reduce Fe^3+^ into Fe^2+^. Moreover, the study of Skemiene et al., 2020 [46], showed that the intravenous injection of cyanidin-3-galactoside and cyaniding-3-glucoside (0.025 mg/kg or 0.05 mg/kg) protected against ischemia-induced caspase activation and necrotic cell death in rat brains, and reduced infarct size. These effects correlated with the capacity of cyanidin-3-glycosides to reduce cytochrome c [46]. Our data revealed that pretreatment with CAPE protected the kidneys from necrotic cell death after 20, 30, and 40 min of ischemia/30 min reperfusion, and completely restored the activity of LDH.

Moreover, our study revealed that CAPE protects the mitochondrial oxidative phosphorylation system from the damage incurred during ischemia/reperfusion. It was most visible when the mitochondria oxidized the Complex I substrates glutamate/malate. The kidney mitochondrial respiration rate (state 3) was clearly increased after the pretreatment of rats with the intravenous injection of CAPE, and there was an obvious increase in mitochondrial Complex I activity. However, there was no protective effect of pretreatment with CAPE on succinate (Complex II)-dependent oxidation. The activity of SDH was partially restored after pretreatment with CAPE, whereas there was no effect on the activity of Complex II+III. It seems that mitochondrial Complex I could evidently be affected during kidney ischemia/reperfusion injury due to the formation of ROS or changes in cardiolipin (our unpublished data), which accounts for roughly 20% of the mitochondrial inner membrane phospholipids [47]. The detachment of cytochrome c from cardiolipin has been shown to be mediated by reactive oxygen species (ROS). We assume that CAPE, acting as a strong antioxidant, might accumulate in the membranes and protect the kidney mitochondria from oxidative stress (ischemia/reperfusion)-induced damage.

It was also demonstrated by Feng et al. that CAPE and its related phenolic compounds could protect the efficiency of oxidative phosphorylation (state 3 increased and state 4 decreased), as shown by the ADP/O ratio [48]. This is in agreement with our data and with our previous data from an in vitro kidney ischemia model [2] that showed an increase in the mitochondrial state 3 respiration (V_ADP_) with glutamate/malate after pretreatment with two doses of CAPE (22 mg/kg and 34 mg/kg) as compared with the untreated group (ischemia/reperfusion).

**Figure 12:**
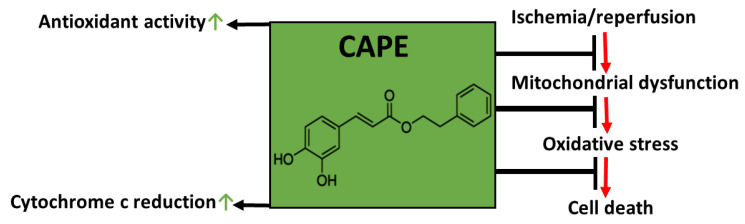
Schematic illustration of our hypothesis.

Thus, our data showed that the diminishing of oxidative stress in the mitochondria after pretreatment with CAPE could explain the improvement of mitochondrial functions after ischemia/reperfusion (Figure 12).

## 5. Conclusions

In this study we investigated whether CAPE has potential protective effects against ischemia/reperfusion-induced kidney damage in an in vivo rat model of warm kidney ischemia. Our main finding is that CAPE (1) possesses antioxidant activity (the reducing properties of CAPE are more pronounced than its antiradical properties); CAPE effectively reduces cytochrome c; (2) protects the glutamate/malate oxidation and Complex I activity; (3) preserves the mitochondrial outer membrane from damage and from the release of cytochrome c; (4) inhibits ROS generation at the Complex II (SDH) F site; (5) diminishes ischemia/reperfusion-induced LDH release and protects from necrotic cell death; and (6) has no protective effects on succinate oxidation or Complex II+III activity, but partially protects SDH from ischemia/reperfusion-induced damage.

In summary, caffeic acid phenethyl ester protects the kidney mitochondria from oxidative stress-induced damage in an in vivo ischemia/reperfusion model, and shows potential as a therapeutic agent for the development of pharmaceutical preparations against oxidative stress-related diseases.

## Figures and Tables

**Table 1:** TEAC values of CAPE.

Method	TEAC
ABTS	0.85 ± 0.07
DPPH	1.65 ± 0.09
FRAP	1.22 ± 0.03
CUPRAC	2.24 ± 0.05

**Table 2:** Effect of ischemia/reperfusion and CAPE on LDH activity in the cytosolic fraction.

	Without CAPE	+22 mg/kg CAPE
Control	0.42 ± 0.01	0.44 ± 0.01
I20/R30	0.29 ± 0.03 *	0.39 ± 0.01 ^#^
I30/R30	0.36 ± 0.02 *	0.42 ± 0.01 ^#^
I40/R30	0.30 ± 0.01 *	0.41 ± 0.02 ^#^
I60/R30	0.38 ± 0.02	0.37 ± 0.03

LDH activity was measured as described in the Materials and Methods section. * *p* < 0.05 vs. the respective control; # *p* < 0.05 vs. ischemia/reperfusion without CAPE. One-way ANOVA followed by a Fisher’s LSD post hoc test were used to compare the effects.

## Data Availability

The data that support the findings of this study are available from the corresponding author upon request.

## Appendix A

**Table A1:** Calibration curve (y = ax + b) data obtained by ABTS and FRAP assays for the tested compounds.

Compound	Concentration Range (mM)	ABTS Method				FRAP Method			
		Slope (a)	Intercept (b)	Correlation (R)	Coefficient of Determination (R^2^)	Slope (a)	Intercept (b)	Correlation (R)	Coefficient of Determination (R^2^)
Trolox	0.25–16	0.0883	+0.0248	0.9995	0.9991	0.1179	+0.0164	0.9992	0.9986
CAPE	0.22–7	0.0753	+0.0697	0.9999	0.9999	0.1443	−0.0388	0.9996	0.9994

**Table A2:** Calibration curve (y = ax + b) data obtained by DPPH and CUPRAC assays for the tested compounds.

Compound	Concentration Range (mM)	DPPH Method				CUPRAC Method			
		Slope (a)	Intercept (b)	Correlation (R)	Coefficient of Determination (R^2^)	Slope (a)	Intercept (b)	Correlation (R)	Coefficient of Determination (R^2^)
Trolox	0.25–32	0.0708	+0.0223	0.9995	0.9991	0.04	−0.0161	0.9994	0.999
CAPE	0.22–14	0.1145	+0.0295	0.9996	0.9993	0.0903	+0.0124	0.9992	0.9986
